# Predictive value of the SLC22A18 protein expression in glioblastoma patients receiving temozolomide therapy

**DOI:** 10.1186/1479-5876-11-69

**Published:** 2013-03-20

**Authors:** Sheng-Hua Chu, Yan-Bin Ma, Dong-Fu Feng, Zhi-Qiang Li, Pu-Cha Jiang

**Affiliations:** 1 Department of Neurosurgery, Shanghai 3rd People's Hospital, School of Medicine, Shanghai Jiao Tong University, 280 Mohe Road, Baoshan District, Shanghai 201900, China; 2 Department of Neurosurgery, Zhongnan Hospital of Wuhan University, Wuhan 430071, China

## Abstract

**Background:**

Our previous study showed that SLC22A18 downregulation and promoter methylation were associated with the development and progression of glioma and the elevated expression of SLC22A18 was found to increase the sensitivity of glioma U251 cells to the anticancer drug 1,3-bis(2-chloroethyl)-1-nitrosourea (BCNU). In this study, we investigated the predictive value of SLC22A18 promoter methylation and protein expression in glioblastoma multiforme (GBM) patients receiving temozolomide (TMZ) therapy.

**Patients and methods:**

SLC22A18 promoter methylation and protein expression were examined by methylation-specific polymerase chain reaction (MSP) and Western blotting respectively, then we compared SLC22A18 promoter methylation and protein expression in tumor cell explants in regard to prediction of TMZ response and survival time of 86 GBM patients.

**Results:**

SLC22A18 promoter methylation was detected in 61 of 86 (71%) samples, whereas 36 of 86 (42%) cases were scored positive for SLC22A18 protein expression. Overall SLC22A18 promoter methylation was significantly related to SLC22A18 protein expression, but a subgroup of cases did not follow this association. Multivariate Cox regression analysis indicated that SLC22A18 protein expression, but not promoter methylation, was significantly correlated with TMZ therapy. SLC22A18 protein expression predicted a significantly shorter overall survival in 51 patients receiving TMZ therapy, whereas no differences in overall survival were observed in 35 patients without TMZ therapy.

**Conclusions:**

These results show that lack of SLC22A18 protein expression is superior to promoter methylation as a predictive tumor biomarker in GBM patients receiving temozolomide therapy.

## Background

Glioblastoma multiforme (GBM) is the most common and lethal glial tumor of the adult brain, accounting for about fifty percent of all gliomas. It is characterized by an aggressive growth pattern, a marked degree of the invasiveness and very poor prognosis. The standard treatment for malignant glioma patients was resection followed by radiotherapy in the past many years. Lately a great lot of researches revealed a statistically significant survival benefit for GBM patients treated with radiotherapy plus temozolomide (TMZ) [1,2]. Consequently, radiotherapy plus concurrent TMZ therapy currently represents the standard of care for newly diagnosed GBM patients [3].

Solute carrier family 22 (organic cation transporter) member 18 (SLC22A18), also known as IMPT1/BWR1A/TSSC5, is located within the human 11p15.5 cluster [4,5]. Blast homology analysis suggests that SLC22A18 is a member of the family of polyspecific transporters and multidrug resistance genes [5]. More recently, SLC22A18 has been shown to be a tumor suppressor candidate and a substrate for RING105 [6]. Structural mutations in SLC22A18 are rare, with isolated reports of point mutations in a breast cancer cell line [7], a rhabdomyosarcoma cell line [5], and Wilms’ tumors and lung tumors [8]. Exonic deletions in Wilms’ tumors and loss of heterozygosity in hepatoblastomas have also been reported [9], indicating that SLC22A18 may play a role in tumorigenesis. We have previously found that SLC22A18 downregulation and promoter methylation were associated with the development and progression of glioma and SLC22A18 represented a candidate biomarker for long-term survival in this disease, suggesting that SLC22A18 is an important tumor suppressor in glioma [10,11]. We have also found that the elevated expression of SLC22A18 was found to increase the sensitivity of glioma U251 cells to the anticancer drug 1,3-bis(2-chloroethyl)-1-nitrosourea (BCNU) [12].

In this study, for the first time we used primary tumor cell explants obtained from GBM surgical specimens instead of tissue samples to investigate SLC22A18 methylation promoter and protein expression of tumor cell. Our data presented show that expression of SLC22A18 protein has a very strong predictive value for TMZ response and survival time in GBM patients.

## Materials and methods

### Study patients

We collected 86 cases of surgically resected GBMs in the period ranging from 2007–2010 at the Department of Neurosurgery, NO.3 People's Hospital Affiliated to Shanghai Jiao Tong University School of Medicine and Zhongnan Hospital of Wuhan University. Informed patient consent and prior approval from the NO.3 People’s Hospital Affiliated to Shanghai Jiao Tong University School of Medicine and Zhongnan Hospital of Wuhan University Ethics Committees (Ethic approval ZNHWHU0389,NTPHSHJTUSM046) was obtained before the clinical materials were used for research purposes. All experiments on humans in the present study were performed in compliance with the Helsinki Declaration. Gadolinium-enhanced MRI performed within 1 week after surgery was used to categorize the surgical results according to the removed tumor proportion, i.e., biopsy, ≤ 50%; partial removal, 50-95%; subtotal removal, 96-99%; total removal, >99%. There were 46 men and 40 women with a mean age of 62.5 years (range from 14 to 78 years). None of the patients had received chemical therapy or radiotherapy prior to surgery. 50 patients received concurrent radiotherapy and chemotherapy (60 Gy and daily TMZ at 75 mg/m^2^; 7 days per week over a 42-day period) after surgery. 30 patients received the adjuvant TMZ after concurrent radio-temozolomide therapy. 36 patients did not receive chemotherapy, out of which 18 had only radiotherapy and 18 did not have further therapy. Characteristics of the patients according to SLC22A18 promoter methylation and protein expression status were shown in Table 1.

**Table 1:** Characteristics of the patients according to SLC22A18 promoter methylation and protein expression status

**Variables**	***N*** **= 86 No. (%)**	**SLC22A18 promoter methylation status, No. (%)**		***P*-value**	**SLC22A18 protein expression, No. (%)**		***P*-value**
		**Methylated *N*** **= 61(71)**	**Unmethylated *N*** **= 25(29)**		**Positive *N*** **= 36(42)**	**Negative *N*** **= 50(58)**	
Gender							
Male	46(53)	36(59)	10(40)	0.11	21(58)	25(50)	0.45
Fmale	40(47)	25(41)	15(60)		15(42)	25(50)	
Age (years)							
<45	13(15)	8(13)	5(20)	0.42	4(11)	9(18)	0.38
≥45	73(85)	53(87)	20(80)		32(89)	41(82)	
Performance status							
<90	39(45)	27(44)	12(48)	0.75	16(44)	23(46)	0.89
≥90	47(55)	34(56)	13(52)		20(56)	27(54)	
Extent of surgery							
Total	45(52)	34(56)	11(44)	0.32	19(67)	26(42)	0.94
Not total	41(48)	27(44)	14(56)		17(33)	24(58)	
MGMT promoter							
Unmethylation	29(34)	18(30)	11(44)	0.20	12(33)	17(34)	0.95
Methylation	57(66)	43(70)	14(56)		24(67)	33(66)	
TMZ therapy							
Yes	50(58)	37(61)	13(52)	0.46	23(64)	27(54)	0.36
No	36(42)	24(39)	12(48)		13(36)	23(46)	

### Primary tumor cell cultures

After removing visible blood vessel and necrotic tissues, surgery specimens were processed for primary cell cultures as described previously [13-15], and analyzed between passages 2 and 6. During resection patches from non-necrotic parts of the tumors were gained, disaggregatd mechanically and transferred into culture flasks, containing growth medium (RPMI-1640, 20% fetal bovine serum, 1% penicillin/streptomycine, 1% glutamine; Gibco Life Technologies, Paisley, Scotland, UK). Three to five culture flasks were set up from each surgery specimen. Cells were then cultured in RPMI-1640 supplemented with 10% fetal bovine serum and 1% glutamine and without antibiotics. The presence of predominantly malignant cells in primary cell cultures was proved by comparative genomic hybridization and/or fluorescent *in situ* hybridization analyses [13]. All cell cultures were periodically checked for Mycoplasma contamination using a Mycoplasma Stain Kit (Sigma-Aldrich, St. Louis, Missouri, USA) [16].

### DNA extraction and methylation-specific polymerase chain reaction

As we’ve previously described [10,17], genomic DNA was extracted from tumor cell cultures or frozen surgery tissues by the digestion with proteinase K (Fisher Scientific, Fairlawn, NJ, USA) using a genomic DNA purification kit (PureGene kit, Gentra Systems, Inc., Minneapolis, MN, USA), and 1 μg genomic DNA was treated with a CpG WIZ DNA Modification Kit (EMD Millipore Corporation, Billerica, MA, USA) to convert unmethylated cytosines to uracil, leaving methylated cytosines unchanged. The modified DNA was diluted in TE buffer (10 mM Tris-Cl, pH 7.5. 1 mM EDTA). SLC22A18 and MGMT promoter methylation analysis was performed by PCR, using bisulfite-treated DNA as template, with specific primers for the methylated (unmodified by bisulfite treatment) and unmethylated (bisulfite modified) gene sequences using the methylation-specific polymerase chain reaction (MSP) method [10,17]. Expression levels were quantified by Quantity One Quantitation software (BioRad) and calculated relative to the controls. The results were confirmed by repeating the bisulfite treatment and MSP assays for all samples.

### Western blot analysis

As we’ve previously described [18-20], tumor cell cultures were washed in ice-cold PBS and lysed in buffer using standard methods. tumor tissues were homogenized in a RIPA lysis buffer. Lysates were cleared by centrifugation (14, 000 rpm) at 4°C for 30 minutes. Protein samples (approximately 40 μg) were separated by SDS-PAGE (15% gel), transferred to PVDF membrane and non-specific binding sites blocked by incubation in 5% non-fat milk for 60 minutes. Membranes were incubated overnight at 4°C with polyclonal anti-SLC22A18 antibody (1:1,000 dilution; Santa Cruz, CA). The membrane was then washed three times with TBST for 10 minutes and probed with HRP-conjugated secondary antibody (at 1:2,000 dilution; Dako, Glostrap, Denmark) for 30 minutes at room temperature. Visualization and quantification were done using the ChemiDoc System (BioRad). Data gained by the densitometric analysis of Western blotting for SLC22A18 were expressed relative (arbitrary units) to the SLC22A18 expressing human neurons (ScienCell Research Laboratories, San Diego, CA, USA) that is included as a positive control in each blot and set arbitrarily as 1. Each assay was performed in triplicate.

### Statistical analyses

Statistical analyses and graphs were performed using the Statistical Package for the Social Sciences (version 16.0, for Windows) (SPSS, Chicago, IL, USA). Quantitative values were expressed as mean ± SD. Statistical differences between groups were examined using the Fisher’s exact test. *P*-values less than 0.05 were considered statistically significant.

## Results

### SLC22A18 promoter methylation and protein expression

**Figure 1:**
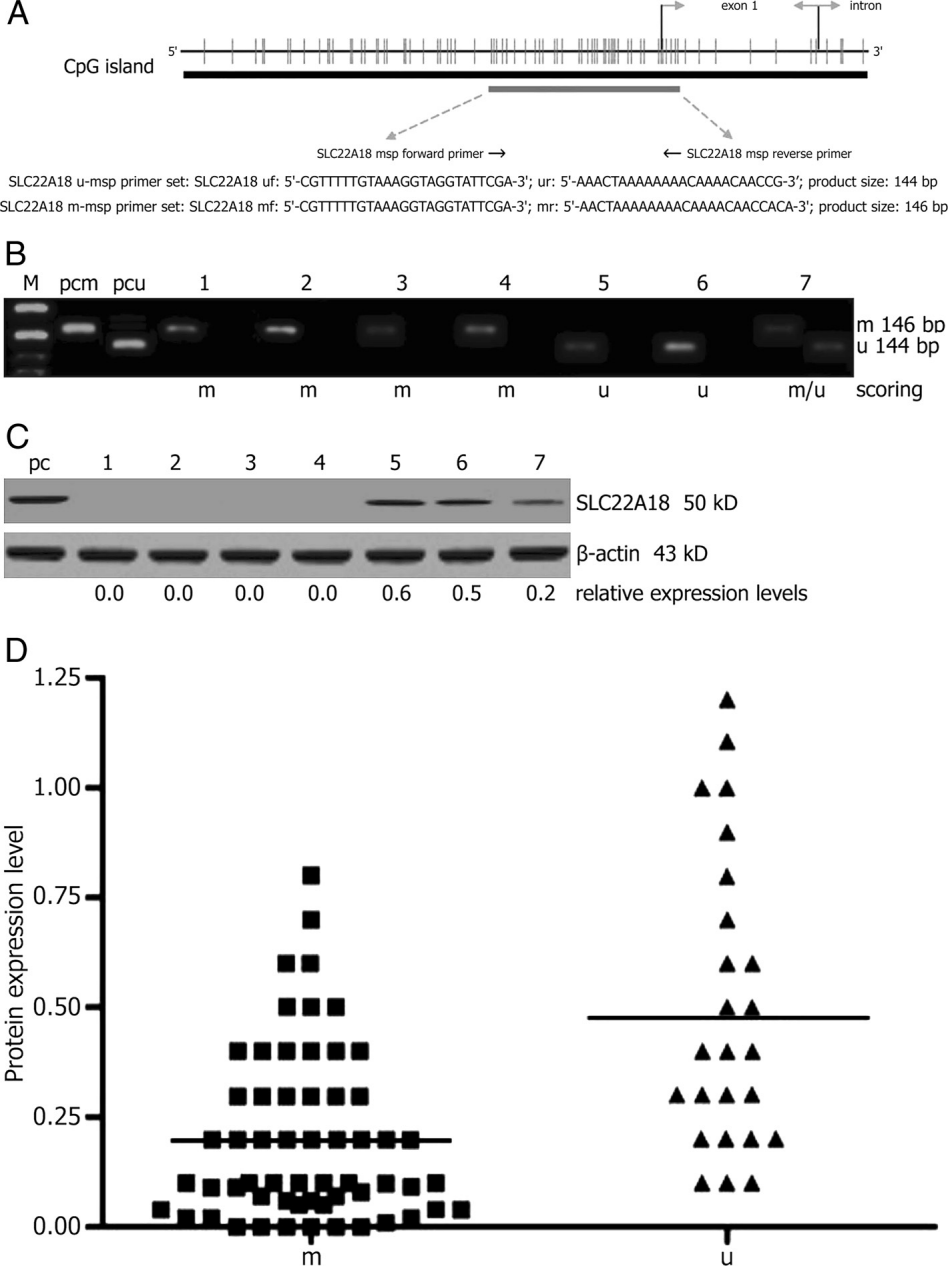
**Analysis of SLC22A18 promoter methylation and protein expression in GBM cell cultures explanted from surgical specimens.** (**A**) CpG island of SLC22A18 gene with location and sequence of methylation-specific polymerase chain reaction (MSP) primers (uf, forward U-MSP primer; ur, reverse U-MSP primer; mf, forward M-MSP primer; mr, reverse M-MSP primer). (**B**) Representative images of MSP analysis of SLC22A18 promoter methylation. Amplification products with specific primers for unmethylated (u) and methylated (m) DNA sequences are indicated, and scoring as unmethylated (u), methylated (m), and mixed (u/m) is shown. Positive controls for unmethylated (pcu) and methylated (pcm) sequences were included. M, size marker. (**C**) Representative images of western blotting analysis of SLC22A18 protein expression, and levels of relative expression were gained by the densitometric analysis of Western blotting compared with the SLC22A18 expressing human neurons included as positive control (pc) and set arbitrarily as 1. β-actin was used as loading control. (**D**) Scatter gram analysis of SLC22A18 protein expression in two subgroups with methylated and unmethylated SLC22A18 promoter sequences.

**Figure 2:**
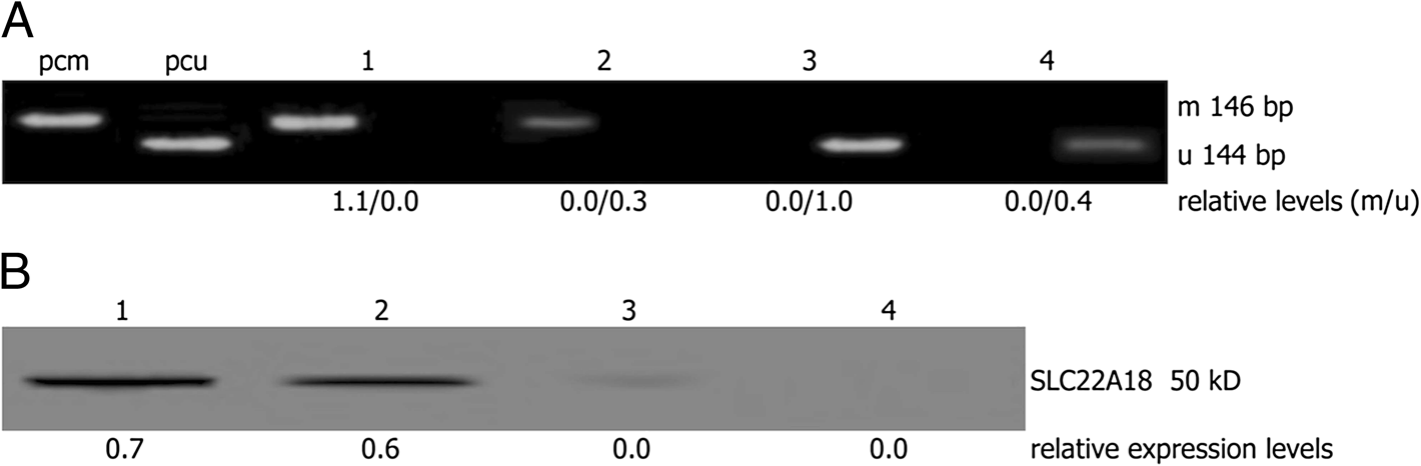
**Discrepancy between SLC22A18 promoter methylation and protein expression.** Representative samples for SLC22A18 promoter methylation and protein expression (1, 2) as well as lack of promoter methylation and protein expression (3, 4) are shown. (**A**) Polyacrylamide gel showing amplification products of unmethylated and methylated DNA sequences by MSP. Positive controls for unmethylated (pcu) and methylated (pcm) sequences were shown. (**B**) Western blot analysis for the corresponding cases.

Primary tumor cell culture was successful *in vitro* in all surgery specimens, allowing SLC22A18 promoter methylation and protein expression analyses. In 61 of 86 (71%) GBM cases methylated SLC22A18 gene promoter sequences were detected. Out of the 61 samples with methylated sequences, 24 samples contained a mixed profile with varying proportions of unmethylated and methylated sequences, whereas the remaining 37 cases (61%) lacked any unmethylated DNA (Figure 1A-B). SLC22A18 promoter was completely unmethylated in 25 of 86 (29%) GBM cases. To investigate the potential influence of tumor cell culture, SLC22A18 promoter methylation was determined in both surgical tissues and the respective tumor cell cultures in 86 cases. SLC22A18 methylation status agreed well in all patients (see Additional file 1: Table S1), and semi-quantification of MSP products for methylated DNA sequences showed a significant correlation (linear regression analysis, *P* < 0.001). In all cell cultures SLC22A18 protein expressions were successfully analyzed by Western blotting (Figure 1C). 36 of the 86 cases (42%) were scored positive for SLC22A18 protein expression based on a visible band and corresponding to a relative expression level > 0.1. Neither SLC22A18 promoter methylation nor protein expression was significantly correlated with any of the clinical features listed in Table 1. The mean SLC22A18 expression levels were significantly lower in tumor cell cultures harboring methylated SLC22A18 promoter sequences when compared with only unmethylated subgroup (*P* < 0.001) (Figure 1D). Therefore, SLC22A18 protein expression was significantly associated with promoter methylation status when analyzed by 2-sided *χ*^2^ test (*P* < 0.001). SLC22A18 protein expression levels in the subgroups were strongly overlapping according to promoter methylation despite this association (Figure 1D). In Figure 2 data of 4 representative samples that did not follow the correlation between SLC22A18 protein expression and promoter methylation status were shown. These findings included cases with exclusively unmethylated promoter sequences but lack of SLC22A18 protein expression as well as high expression of SLC22A18 protein, despite completely methylated promoter.

### Relationship between SLC22A18 status and overall survival of GBM patients with/without TMZ therapy

**Figure 3:**
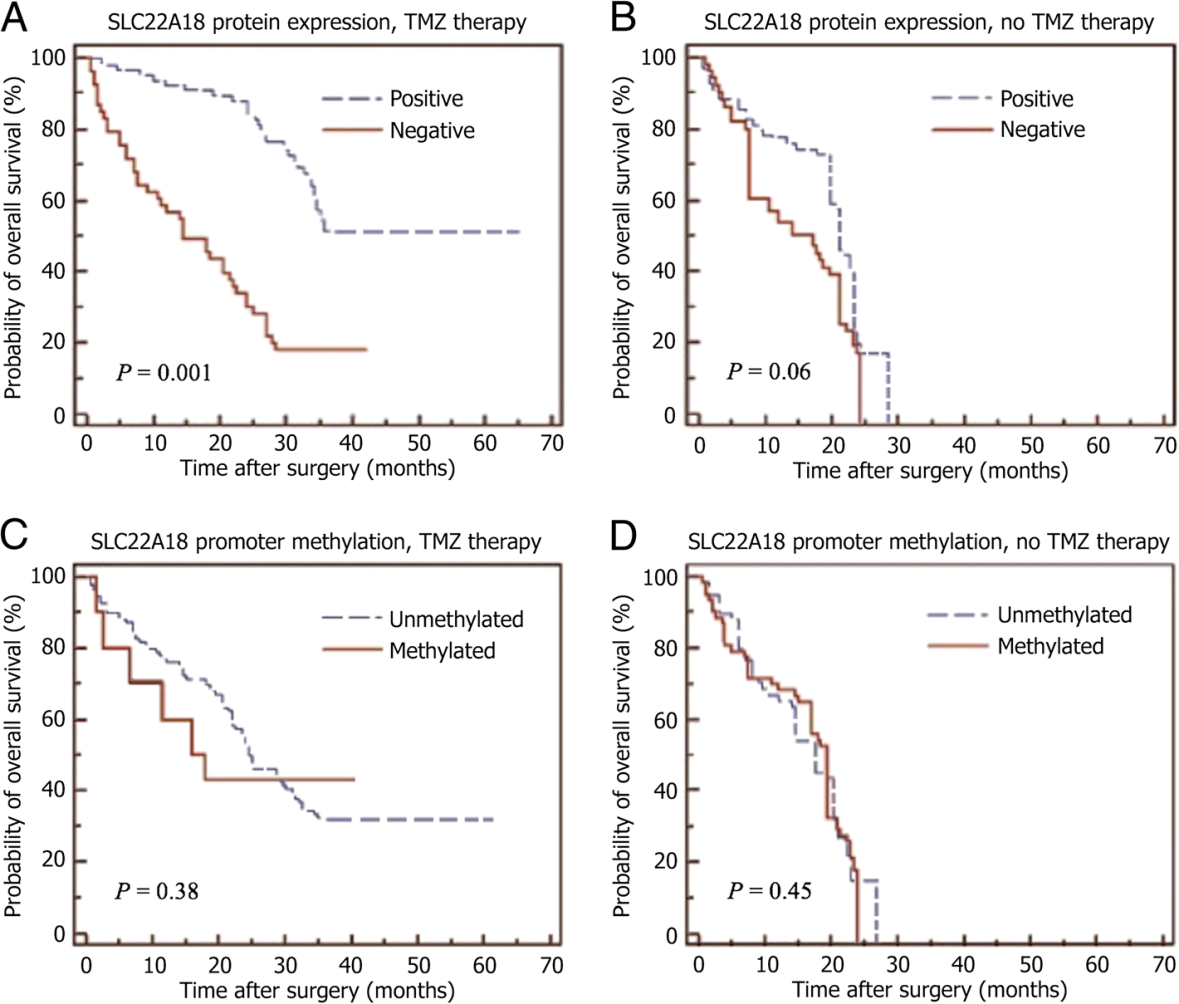
**SLC22A18 promoter methylation status and protein expression when compared with overall survival time of cases.** Results of Kaplan-Meier analyses of overall survival time according to SLC22A18 protein expression (**A** and **B**) or promoter methylation (**C** and **D**) of GBM patients with TMZ therapy (**A** and **C**) and without TMZ therapy (**B** and **D**) are shown.

58 (67%) of 86 cases had died (34 of 35 cases without TMZ treatment and 24 of 51 cases with TMZ treatment) at a median follow-up time of 27.1 months (95% CI 25.8-30.2). By univariate analyses the overall survival of GBM patients was compared to SLC22A18 protein expression or promoter methylation status (Table 2). In the univariate analysis lack of SLC22A18 protein expression (hazard ratio [HR] of death 2.14, 95% CI 1.24-3.72; *P* = 0.01), but not promoter methylation status (HR of death 0.64, 95% CI 0.32-1.15; *P* = 0.13), was significantly connected with shorter overall survival in addition to well-known prognostic factors such as age, performance status, extent of surgery, MGMT promoter methylation and TMZ therapy. Besides age, performance status, extent of surgery, MGMT promoter methylation and TMZ therapy, only lack of SLC22A18 protein expression, but not SLC22A18 promoter methylation status, remained as an independent prognostic factor (HR of death 2.11, 95% CI 1.09-3.77; *P* = 0.02) in multivariate Cox regression analysis (Table 3). An interaction term, the product of SLC22A18 parameter and TMZ therapy, was incorporated into the multivariate Cox regression models to evaluate a possible interaction between the SLC22A18 parameters and TMZ treatment. These findings showed a statistically significant association between SLC22A18 protein expression and TMZ treatment (*P* = 0.004), whereas in the case of SLC22A18 promoter methylation this interaction was not significant. Because the interaction term of SLC22A18 protein expression and TMZ therapy was significant, we studied the relationship between SLC22A18 protein expression and overall survival time in TMZ therapy and no TMZ therapy subgroups (Table 4, Figure 3A and B). In patients with TMZ therapy, SLC22A18 protein expression was significantly associated with longer overall survival time (HR of death 5.65, 95% CI 1.79-17.45; *P* = 0.002). Conversely, no differences in overall survival time according to SLC22A18 protein expression (HR of death 1.12, 95% CI 0.56-2.28; *P* = 0.62) were shown in patients without TMZ therapy. A similar study using the subgroups according to promoter methylation status showed no significant differences with overall survival time in the TMZ therapy subgroup (Figure 3C and D).

**Table 2:** Univariate survival analysis

**Variable**	**HR**	**95% CI**	***P *value**
Gender	0.75	0.38-1.32	0.24
Age	1.12	1.02-1.14	< 0.001
Performance status	0.98	0.92-1.02	< 0.001
Extent of surgery	0.14	0.10-0.28	< 0.001
MGMT methylation	1.25	1.12-1.32	0.006
TMZ therapy	0.24	0.12-0.41	< 0.001
SLC22A18 promoter methylation	0.64	0.32-1.15	0.13
SLC22A18 protein expression	2.14	1.24-3.72	0.01

**Table 3:** Multivariate survival analysis

	**Promoter methylation**			**Protein expression**		
	**HR**	**95% CI**	***P *value**	**HR**	**95% CI**	***P *value**
Variable without interaction term						
Gender	0.83	0.41-1.52	0.44	0.95	0.46-1.76	0.86
Age	1.10	1.01-1.20	0.002	1.10	1.01-1.12	0.002
Performance status	0.98	0.92-1.02	0.001	0.98	0.92-1.02	0.001
Extent of surgery	0.14	0.08-0.22	< 0.001	0.14	0.08-0.24	< 0.001
MGMT promoter	1.32	1.11-1.56	0.012	1.32	1.11-1.55	0.012
TMZ therapy	0.29	0.14-0.50	< 0.001	0.29	0.14-0.49	< 0.001
Promoter methylation	0.62	0.31-1.19	0.15			
Protein expression				2.11	1.09-3.77	0.02
Variable with interaction terms						
Gender	0.83	0.41-1.55	0.46	1.02	0.50-1.87	0.95
Age	1.10	1.01-1.20	0.002	1.10	1.01-1.22	0.001
Performance status	0.98	0.92-1.03	0.002	0.98	0.92-1.02	0.001
Extent of surgery	0.14	0.08-0.22	< 0.001	0.14	0.08-0.34	< 0.001
MGMT promoter	1.32	1.11-1.57	0.018	1.32	1.11-1.55	0.012
TMZ therapy	0.32	0.11-0.83	0.02	0.11	0.03-0.25	< 0.001
Promoter methylation	0.67	0.59-2.91	0.33			
Protein expression				1.06	0.45-2.11	0.45
Promoter methylation × TMZ therapy			0.83			
Protein expression × TMZ therapy						0.004

**Table 4:** Multivariate survival analysis in subgroups of cases regarding TMZ therapy

**Variable**	**TMZ therapy**			**No TMZ therapy**		
	HR	95% CI	*P* value	HR	95% CI	*P* value
Gender	0.45	0.12-1.25	0.08	1.82	0.76-4.06	0.12
Age	1.12	1.01-1.16	0.005	1.10	0.98-1.14	0.03
Performance status	0.97	0.90-1.02	0.006	0.99	0.92-1.04	0.01
Extent of surgery	1.05	0.98-1.12	0.003	1.02	0.96-1.18	0.01
MGMT promoter	5.85	1.86-18.02	0.001	1.25	0.62-2.34	0.74
SLC22A18 Protein expression	5.65	1.79-17.45	0.002	1.12	0.56-2.28	0.62

## Discussion

We have previously found that SLC22A18 protein expression was significantly decreased in human gliomas compared to the adjacent normal brain tissues. SLC22A18 protein expression was significantly lower in gliomas which recurred within six months after surgery than gliomas which did not recur within six months. Multivariate Cox regression analysis indicated that SLC22A18 expression level was an independent survival prognostic factor for patients with glioma. SLC22A18 promoter methylation was detected in 50% of the gliomas, but not in the adjacent normal tissues of any patient. SLC22A18 expression was significantly decreased in gliomas with SLC22A18 promoter methylation, compared to gliomas without methylation. The SLC22A18 promoter was methylated in U251 cells and treatment with the demethylating agent 5-aza-2-deoxycytidine increased SLC22A18 expression and reduced cell proliferation. Stable overexpression of SLC22A18 inhibited growth and adherence, induced apoptosis *in vitro* and reduced *in vivo* tumor growth of U251 cells [10,11,21]. We have also found that elevated expression of SLC22A18 increased the sensitivity of U251 glioma cells to BCNU [12]. Recently we have found that by increasing the expression of SLC22A18 hydroxyapatite nanoparticles could inhibit the growth of human glioma cells *in vitro* and *in vivo*[18].

In the present study, we have studied the relationship between SLC22A18 promoter methylation and protein expression and the association of these two parameters with TMZ treatment as well as overall survival time of GBM patients. We determined SLC22A18 protein expression by Western blot analysis in primary tumor cells derived from the surgical specimens instead of tissue sections or tumor extracts in order to focus completely on the malignant cell compartment. To avoid problems arising from the tissue fixation and paraffin embedding, we used cell cultures-derived DNA for detection of SLC22A18 promoter methylation satus; and MSP was performed comparably from tumor cells and snap-frozen tissue in a subgroup of tumors, confirming the presence of methylated SLC22A18 promoter sequences in all cases. During short-term *in vitro* cultivation of tumor cells, the observations also exclude possible changes in the SLC22A18 promoter methylation status. Using this new method, we demonstrate that SLC22A18 protein expression is a strong important predictive marker for the response of TMZ therapy and the related survival benefit. our results showed SLC22A18 protein expression was associated with longer overall survival time. Especially in patients with TMZ therapy, SLC22A18 protein expression was significantly associated with longer overall survival time, and in patients without TMZ therapy no significant differences in overall survival time according to SLC22A18 protein expression were shown. The reasons for the discrepancy of SLC22A18 expression in different tumor patients and a patient subgroup are unclear so far. Thus, future studies with larger sample sizes should be done to confirm this trend. SLC22A18 protein expression was of superior predictive value in GBM patients in our study despite a strong association between SLC22A18 promoter methylation and protein expression. These findings show that reliable SLC22A18 protein detection approach has to be developed to identify GBM patients likely to benefit from TMZ treatment. Consistent with this idea [10], the SLC22A18 parameters showed significant correlation in this study, confirming that SLC22A18 promoter methylation is a major factor regulating SLC22A18 protein expression. However, as obvious from the scatter grams (Figure 1D) and depicted by selected patients (Figure 2), there are some tumor cell samples not following this association. This shows that SLC22A18 protein is still expressed in a subgroup of tumors with SLC22A18 promoter methylation and vice versa. The reasons for the discrepancy between SLC22A18 expression and promoter methylation in a patient subgroup are unclear so far. Our results show that epigenetic silencing via promoter methylation is an important but obviously not always decisive factor regulating SLC22A18 expression in GBM cells. With respect to therapy response and overall survival, SLC22A18 protein expression proved to be of significantly higher predictive power when compared with promoter methylation status in our samples. Our attempts to analyze SLC22A18 expression by immunohistochemistry in selected patient samples used in this study were inconclusive, and immunostaining neither correlated with SLC22A18 expression nor promoter methylation detected by Western blot, even when using identical antibodies (data not shown). Our data based on tumor cell explants and Western blot analysis turn out the high quality of SLC22A18 expression as a predictive marker for TMZ response in patients with GBM.

## Conclusions

Collectively, we analyzed the influence of SLC22A18 status on the response of TMZ treatment and survival time of GBM patients by using a completely novel approach. Evaluation of SLC22A18 protein levels in GBM cell cultures derived from surgical specimens was proved to be a strongly predictive indicator for TMZ therapy-mediated survival time benefit with superior information power as compared to the MSP analysis of SLC22A18 promoter methylation. Accordingly, reproducible and reliable SLC22A18 protein detection methods have to be developed and tested with respect to their predictive value for the response of TMZ treatment in prospective studies involving more GBM patients.

## Abbreviations

SLC22A18: Solute carrier family 22 (organic cation transporter) member 18; RPMI: Roswell park memorial institute; PBS: Phosphate-buffered saline; SDS: Sodium dodecyl sulfate; MSP: Methylation-specific polymerase chain reaction.

## Competing interests

The authors declare that they have no competing interests.

## Authors’ contributions

SHC, ZQL and PCJ carried out the laboratory analysis. SHC, YBM and DFF participated in the design of the study and drafted the manuscript. SHC, ZQL and PCJ conceived of the study, and participated in its design and coordination and helped to draft the manuscript. All authors read and approved the final manuscript.

## Supplementary Material

Additional file 1: Table S1 SLC22A18 promoter methylation of DNA extracted from primary GBM cell cultures and surgical tissues. Click here for file

## References

[B1] Stupp R Mason WP van den Bent MJ Weller M Fisher B Taphoorn MJ Belanger K Brandes AA Marosi C Bogdahn U Curschmann J Janzer RC Ludwin SK Gorlia T Allgeier A Lacombe D Cairncross JG Eisenhauer E Mirimanoff RO European organisation for research and treatment of cancer brain tumor and radiotherapy groups; national cancer institute of Canada clinical trials group: radiotherapy plus concomitant and adjuvant temozolomide for glioblastoma N Engl J Med 2005 352 987 996 10.1056/NEJMoa043330 15758009

[B2] Stupp R Hegi ME Gilbert MR Chakravarti A Chemoradiotherapy in malignant glioma: standard of care and future directions J Clin Oncol 2007 25 4127 4136 10.1200/JCO.2007.11.8554 17827463

[B3] Grossman SA Ye X Piantadosi S Desideri S Nabors LB Rosenfeld M Fisher J NABTT CNS consortium: survival of patients with newly diagnosed glioblastoma treated with radiation and temozolomide in research studies in the United States Clin Cancer Res 2010 16 2443 2449 10.1158/1078-0432.CCR-09-3106 20371685 PMC2861898

[B4] Lee MP D Reeves C Schmitt A Su K Connors TD Hu RJ Brandenburg S Lee MJ Miller G Feinberg AP Somatic mutation of TSSC5, a novel imprinted gene from human chromosome 11p15.5 Cancer Res 1998 58 4155 4159 9751628

[B5] Schwienbacher C Sabbioni S Campi M Veronese A Bernardi G Menegatti A Hatada I Mukai T Ohashi H Barbanti-Brodano G Croce CM Negrini M Transcriptional map of 170-kb region at chromosome 11p15.5: identification and mutational analysis of the BWR1A gene reveals the presence of mutations in tumor samples Proc Natl Acad Sci USA 1998 95 3873 3878 10.1073/pnas.95.7.3873 9520460 PMC19930

[B6] Yamada HY Gorbsky GJ Tumor suppressor candidate TSSC5 is regulated by UbcH6 and a novel ubiquitin ligase RING105 Oncogene 2006 25 1330 1339 10.1038/sj.onc.1209167 16314844 PMC2713668

[B7] Gallagher E Mc Goldrick A Chung WY Mc Cormack O Harrison M Kerin M Dervan PA Mc Cann A Gain of imprinting of SLC22A18 sense and antisense transcripts in human breast cancer Genomics 2006 88 12 17 10.1016/j.ygeno.2006.02.004 16624517

[B8] Schwienbacher C Angioni A Scelfo R Veronese A Calin GA Massazza G Hatada I Barbanti-Brodano G Negrini M Abnormal RNA expression of 11p15 imprinted genes and kidney developmental genes in Wilms’ tumor Cancer Res 2000 60 1521 1525 10749116

[B9] Albrecht S Hartmann W Houshdaran F Koch A Gärtner B Prawitt D Zabel BU Russo P Von Schweinitz D Pietsch T Allelic loss but absence of mutations in the polyspecific transporter gene BWR1A on 11p15.5 in hepatoblastoma Int J Cancer 2004 111 627 632 10.1002/ijc.20280 15239143

[B10] Chu SH Feng DF Ma YB Zhang H Zhu ZA Li ZQ Jiang PC Promoter methylation and downregulation of SLC22A18 are associated with the development and progression of human glioma J Transl Med 2011 9 156 10.1186/1479-5876-9-156 21936894 PMC3184631

[B11] Chu SH Ma YB Feng DF Zhang H Zhu ZA Li ZQ Jiang PC Correlation of low SLC22A18 expression with poor prognosis in patients with glioma J Clin Neurosci 2012 19 95 98 10.1016/j.jocn.2011.04.032 22153794

[B12] Chu SH Ma YB Feng DF Zhang H Qiu JH Zhu ZA Elevated expression of solute carrier family 22 member 18 increases the sensitivity of U251 glioma cells to BCNU Oncol Lett 2011 2 1139 1142 22848278 10.3892/ol.2011.371 PMC3406540

[B13] Spiegl-Kreinecker S Pirker C Filipits M Lötsch D Buchroithner J Pichler J Silye R Weis S Micksche M Fischer J Berger W O6-Methylguanine DNA methyltransferase protein expression in tumor cells predicts outcome of temozolomide therapy in glioblastoma patients Neuro Oncol 2010 12 28 36 10.1093/neuonc/nop003 20150365 PMC2940563

[B14] Spiegl-Kreinecker S Pirker C Marosi C Buchroithner J Pichler J Silye R Fischer J Micksche M Berger W Dynamics of chemosensitivity and chromosomal instability in recurrent glioblastoma Br J Cancer 2007 96 960 969 10.1038/sj.bjc.6603652 17342095 PMC2360110

[B15] Peigñan L Garrido W Segura R Melo R Rojas D Cárcamo JG San Martín R Quezada C Combined use of anticancer drugs and an inhibitor of multiple drug resistance-associated protein-1 increases sensitivity and decreases survival of glioblastoma multiforme cells *in vitro* Neurochem Res 2011 36 1397 1406 10.1007/s11064-011-0464-8 21544552

[B16] Lu DY Yeh WL Huang SM Tang CH Lin HY Chou SJ Osteopontin increases heme oxygenase-1 expression and subsequently induces cell migration and invasion in glioma cells Neuro Oncol 2012 14 1367 1378 10.1093/neuonc/nos262 23074199 PMC3480271

[B17] Chu SH Ma YB Feng DF Zhang H Zhu ZA Li ZQ Jiang PC Upregulation of SATB1 is associated with the development and progression of glioma J Transl Med 2012 10 149 10.1186/1479-5876-10-149 22839214 PMC3492129

[B18] Chu SH Feng DF Ma YB Li ZQ Hydroxyapatite nanoparticles inhibit the growth of human glioma cells *in vitro* and *in vivo* Int J Nanomed 2012 7 3659 3666 10.2147/IJN.S33584 PMC3414202 22888225

[B19] Chu SH Feng DF Zhang H Chen ET Duan ZX Li XY Li J Ma YB Zhu ZA Qiu JH c-Met-targeted RNA interference inhibits growth and metastasis of glioma U251 cells *in vitro* J Neurooncol 2009 93 183 189 10.1007/s11060-008-9772-5 19165419

[B20] Chu SH Ma YB Feng DF Zhang H Qiu JH Zhu ZA c-Met antisense oligodeoxynucleotides increase sensitivity of human glioma cells to paclitaxel Oncol Rep 2010 24 189 194 20514461 10.3892/or_00000845

[B21] Chu SH Ma YB Feng DF Zhang H Qiu JH Zhu ZA Effect of 5-Aza-2’-deoxycytidine on SLC22A18 in glioma U251 cells Mol Med Report 2012 5 138 141 10.3892/mmr.2011.620 21993522

