# Supplementary material for: Predictive value of the SLC22A18 protein expression in glioblastoma patients receiving temozolomide therapy
Source: J Transl Med. 2013 Mar 20;11:69. doi: 10.1186/1479-5876-11-69 (PMC3610152; doi:10.1186/1479-5876-11-69)
Supplement: Additional file 1: Table S1 — SLC22A18 promoter methylation of DNA extracted from primary GBM cell cultures and surgical tissues. [file 1479-5876-11-69-S1.doc]

**Supplementary Table 1.** SLC22A18 promoter methylation of DNA extracted from primary GBM cell cultures and surgical tissues

| Case | Methylated SLC22A18 promoter sequences | |
| --- | --- | --- |
|  | Primary cell culture | Surgical tissue |
| 1 | 0.6 | 0.2 |
| 2 | 0.9 | 0.3 |
| 3 | 0 | 0 |
| 4 | 1.2 | 0.8 |
| 5 | 0 | 0 |
| 6 | 0 | 0 |
| 7 | 0.5 | 0.2 |
| 8 | 0.7 | 0.8 |
| 9 | 0.4 | 0.3 |
| 10 | 1.6 | 0.9 |
| 11 | 0.3 | 0.2 |
| 12 | 0 | 0 |
| 13 | 1.8 | 1.2 |
| 14 | 0 | 0 |
| 15 | 0.8 | 0.4 |
| 16 | 0.7 | 0.9 |
| 17 | 2.0 | 1.0 |
| 18 | 1.2 | 0.8 |
| 19 | 0 | 0 |
| 20 | 0.9 | 0.5 |
| 21 | 0.7 | 0.4 |
| 22 | 0.8 | 0.3 |
| 23 | 0 | 0 |
| 24 | 0 | 0 |
| 25 | 0 | 0 |
| 26 | 0 | 0 |
| 27 | 0.8 | 0.5 |
| 28 | 0.9 | 0.4 |
| 29 | 0.6 | 0.3 |
| 30 | 1.6 | 0.8 |
| 31 | 0.2 | 0.1 |
| 32 | 1.0 | 0.8 |
| 33 | 1.6 | 1.0 |
| 34 | 0 | 0 |
| 35 | 0.5 | 0.3 |
| 36 | 1.2 | 0.8 |
| 37 | 1.8 | 1.2 |
| 38 | 1.2 | 0.8 |
| 39 | 1.5 | 1.0 |
| 40 | 0.9 | 0.4 |
| 41 | 0.6 | 0.3 |
| 42 | 0.8 | 0.3 |
| 43 | 0 | 0 |
| 44 | 1.2 | 0.8 |
| 45 | 0 | 0 |
| 46 | 0.5 | 0.1 |
| 47 | 0 | 0 |
| 48 | 1.3 | 0.8 |
| 49 | 0.5 | 0.3 |
| 50 | 1.6 | 1.3 |
| 51 | 0.3 | 0.2 |
| 52 | 0 | 0 |
| 53 | 1.8 | 1.2 |
| 54 | 0.7 | 0.8 |
| 55 | 0.8 | 0.3 |
| 56 | 0 | 0 |
| 57 | 2.0 | 1.4 |
| 58 | 1.2 | 0.6 |
| 59 | 0 | 0 |
| 60 | 1.3 | 0.9 |
| 61 | 0.7 | 0.8 |
| 62 | 0.9 | 0.4 |
| 63 | 0 | 0 |
| 64 | 1.2 | 0.8 |
| 65 | 0 | 0 |
| 66 | 0.6 | 0.3 |
| 67 | 0 | 0 |
| 68 | 0.6 | 0.8 |
| 69 | 0.5 | 0.3 |
| 70 | 1.7 | 1.5 |
| 71 | 0.3 | 0.2 |
| 72 | 0 | 0 |
| 73 | 1.7 | 1.0 |
| 74 | 0.8 | 1.0 |
| 75 | 0.9 | 0.6 |
| 76 | 0 | 0 |
| 77 | 1.9 | 1.3 |
| 78 | 1.4 | 0.9 |
| 79 | 0 | 0 |
| 80 | 1.0 | 0.6 |
| 81 | 0.7 | 0.3 |
| 82 | 0.9 | 0.4 |
| 83 | 0 | 0 |
| 84 | 1.2 | 0.9 |
| 85 | 0 | 0 |
| 86 | 1.0 | 0.8 |
